# Exploring Interrelations Between Person-Centered Care and Quality of Life Following a Transition Into Long-Term Residential Care: A Meta-Ethnography

**DOI:** 10.1093/geront/gnac027

**Published:** 2022-02-17

**Authors:** Megan Davies, Franziska Zúñiga, Hilde Verbeek, Michael Simon, Sandra Staudacher

**Affiliations:** Institute of Nursing Science, Department of Public Health, University of Basel, Basel, Switzerland; Department of Health Services Research, Care and Public Health Research Institute, Maastricht University, Maastricht, The Netherlands; Institute of Nursing Science, Department of Public Health, University of Basel, Basel, Switzerland; Department of Health Services Research, Care and Public Health Research Institute, Maastricht University, Maastricht, The Netherlands; Institute of Nursing Science, Department of Public Health, University of Basel, Basel, Switzerland; Institute of Nursing Science, Department of Public Health, University of Basel, Basel, Switzerland; Department of Health Services Research, Care and Public Health Research Institute, Maastricht University, Maastricht, The Netherlands

**Keywords:** Aged care, Care homes, Well-being

## Abstract

**Background and Objectives:**

Globally, a culture change in long-term residential care (LTRC) moving toward person-centered care (PCC) has occurred in an attempt to improve resident quality of life (QoL). However, a clear understanding of how different aspects contributing to a PCC approach are interrelated with resident QoL is still lacking. This review explores interrelating aspects between PCC and QoL in LTRC using qualitative synthesis.

**Research Design and Methods:**

Ten relevant primary studies were identified from a search of interdisciplinary research databases providing qualitative information. Studies were critically reviewed for key themes and concepts by the research team. We used a meta-ethnography approach to inductively interpret findings across multiple studies and reinterpreted the information using a constructivist approach.

**Results:**

We identified 5 second-order constructs sharing commonalities suggesting interrelations between PCC and QoL: (a) maintaining dignity, autonomy, and independence; (b) knowing the whole person; (c) creating a “homelike” environment; (d) establishing a caring culture; and (e) integrating families and nurturing internal and external relationships. Synthesis translation led to the following third-order constructs: (a) personalizing care within routines, (b) optimizing resident environments, and (c) giving residents a voice.

**Discussion and Implications:**

There are many interrelating aspects of PCC and QoL following a permanent transition into LTRC, but successful implementation of PCC, which enhances QoL, presents challenges due to organizational routines and constraints. However, by prioritizing resident voices to include their needs and preferences in care, QoL can be supported following a transition into LTRC.

## Background and Objectives

Levels of daily health care requirements for older adults due to worsening chronic illness, multimorbidities, acute illness, or deterioration of mental health have increased in an aging population (Leichsenring, 2004; World Health Organization [WHO], 2015). This often requires constant and complex care, increasing the necessity of long-term residential care (LTRC) in later life (Johri et al., 2003; Robison et al., 2012). For the purpose of this review, LTRC is used as an umbrella term for institutional environments providing care to older adults residing in this setting on a permanent (24/7) basis. This includes, for example, nursing homes and care homes, as outlined by Moore et al. (2019). Older people show a preference to remain in their own homes, among other things due to negative views of care in LTRC. A culture change movement in LTRC over the last decades has aimed to alter such negative perceptions of a permanent transition into LTRC (Meyer & Owen, 2008). A key element of this culture change movement was a move toward person-centered care (PCC) in an attempt to focus more on individual quality of life (QoL) than biomedical markers of health as a key measure to determine a successful move into LTRC (Musich et al., 2018).

### A Culture Change in LTRC

LTRC facilities provide both medical services and a home environment for older adults (WHO, 2015). By using a more social model of care, residents become the central focus of care and services provided rather than using a “one-size-fits-all” strategy, which promotes resident autonomy and QoL (Crandall et al., 2007; Zimmerman et al., 2014). By adjusting care provisions, the requirements of each resident’s specific circumstances are met during a transition from home to a new LTRC environment (Kane et al., 2004). As a result, a key concept in this change in culture is PCC, which emphasizes the importance of resident well-being and QoL rather than focusing on more medically driven outcome measures previously used in health care for the older population (McCance et al., 2011; Nolan, 2001).

### PCC as a Concept in LTRC

PCC is an overarching term, which takes a holistic approach to care, “whole well-being,” which includes the context, preferences, beliefs, and experiences of an individual and emphasizes living well now above living longer. As a concept, PCC has been outlined using various terms depending on the researcher and with the context in mind, for example, “patient-centered care,” “integrated care,” “resident-centered care,” or “relationship-centered care.” However, despite the varied terms used, the general concept intends on placing core values and resident choice at the center of the care structure (McCance et al., 2011). McCance et al. (2011) define PCC as “an approach to practice established through the formation and fostering of therapeutic relationships between all care providers, patients and others significant to them in their lives” (McCance et al., 2011). A successful PCC culture is developed through strong collaboration between multiple actors, including LTRC staff, external medical professionals, such as GPs, and family members (Dewing & McCormack, 2017; Koren, 2010; Tsakitzidis et al., 2017). A person-centered organizational culture creates a more positive experience for residents following a transition into LTRC, improving self-efficacy and resilience (Bradshaw et al., 2012; Poey et al., 2017; Terada et al., 2013). Although the need for PCC is acknowledged, a lack of clarity on how to put PCC elements into practice and how it works in different contexts and for different individuals has been reported; therefore, a gap between “the rhetoric and the reality” of PCC largely remains (Berntsen et al., 2019).

### QoL in LTRC

Resident QoL has been acknowledged as an overall outcome of “healthy aging” in LTRC (Hughes & Moore, 2012). QoL is defined by the WHO using four domains: (a) physical health, (b) social relationships, (c) psychological health, and (d) environment (WHO, 2015, 2019). While QoL has previously focused on physical health, the importance of individual perceptions based on context, social situations, and spiritual needs are now acknowledged (Pinto et al., 2017). Despite definitions, QoL means different things to different people and is hard to quantify, particularly in the older population where each lived experience is different depending on environment, physical health, and cognitive state (Halvorsrud & Kalfoss, 2007; Levasseur et al., 2009). QoL should itself be considered a person-centered concept (Halvorsrud & Kalfoss, 2007).

LTRC research increasingly aims to understand resident QoL (Post, 2014), focusing on organizational and cultural factors influencing resident experiences (Rahman & Schnelle, 2008; White-Chu et al., 2009). QoL in LTRC should be addressed during the whole trajectory of a resident’s stay, from point of entry onward (Hjaltadóttir & Gustafsdottir, 2007; Moore et al., 2019). Adjustments, such as facilitating personalization of resident rooms for continuity, must therefore be made within LTRC depending on individual circumstances to improve resident QoL and ease the transition into LTRC (Bradshaw et al., 2012; Kane et al., 2004).

LTRC facilities are working to assure practices enhancing QoL; however, resident QoL in LTRC still fails to be adequately measured with a focus on avoiding adverse events rather than accounting for individual experiences to promote resident well-being and QoL (Carr & Higginson, 2001; McGilton et al., 2012). This is particularly the case in residents with cognitive decline or those living with dementia, who are often represented in studies by a proxy, such as staff members (Usman et al., 2019; Wilhelmson, 2005).

### Aims and Objectives

Although PCC as an overall concept is understood to enhance resident QoL, there is not yet a clear understanding of how different aspects contributing to a PCC approach are interrelated with resident QoL. Furthermore, additional insight from the perspectives and experiences of residents is required to fully understand potential interrelations between PCC and QoL (Edvardsson. et al., 2019; Kane, 2003; Klapwijk et al., 2016; Roberts & Ishler, 2017). This review article therefore aims to explore interrelations between PCC and QoL following a permanent transition into LTRC from resident perspectives using qualitative synthesis.

## Research Design and Methods

A meta-ethnography was undertaken as outlined by Noblit and Hare (1988), which synthesizes qualitative information to explore a phenomenon within a real-life context, in this case, LTRC. Meta-ethnography allows inductive and interpretive synthesis. This in-depth analysis explores and explains collectively shared understandings as well as contradictions between studies, providing new insights and offering a single wider conclusion based on the multiple conclusions observed by the author (Tong et al., 2012). The meta-ethnography followed seven phases from inception to completion (Noblit & Hare, 1988).

### Search Strategy

#### Phases 1 and 2

**Figure 1.**
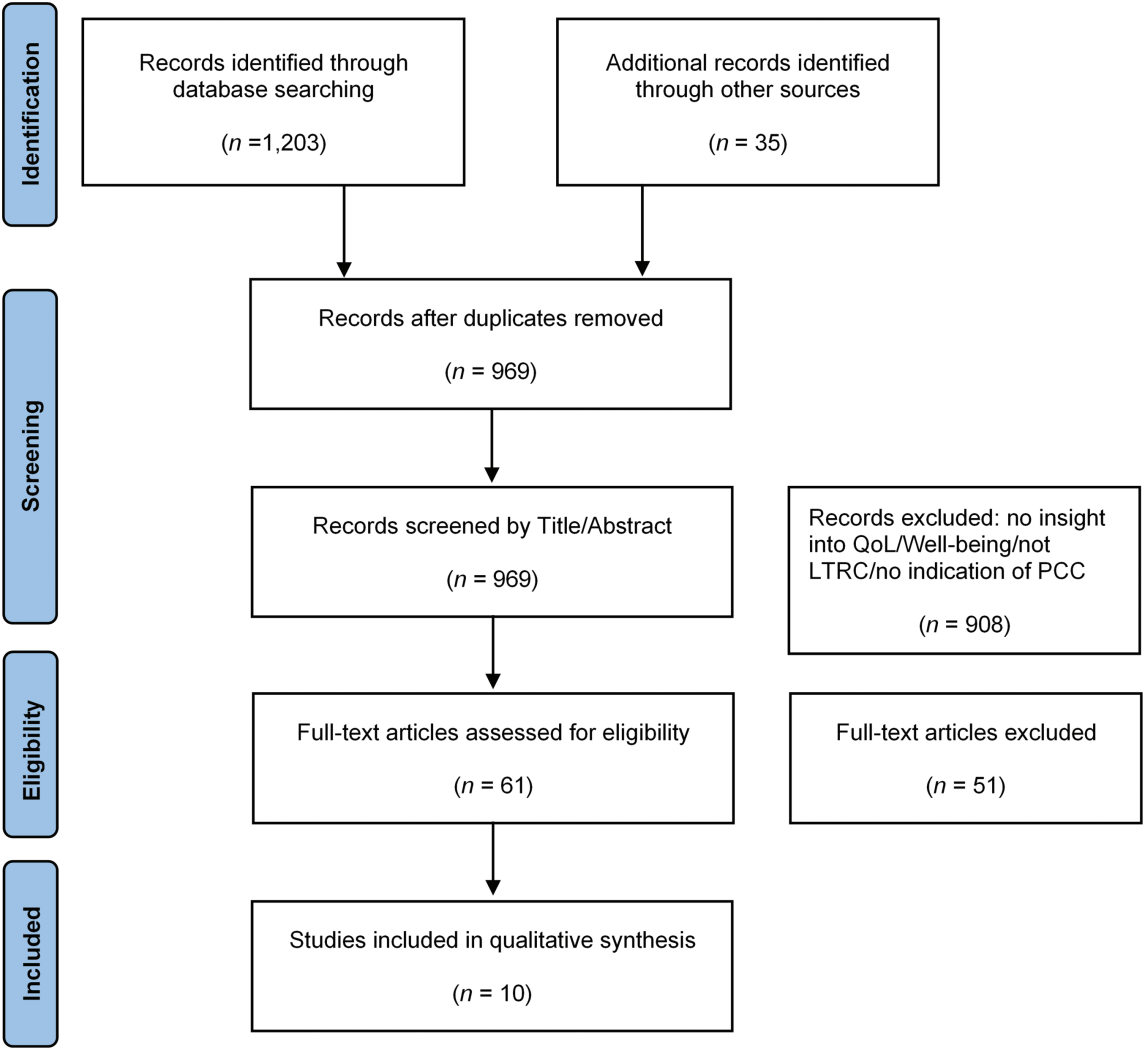
PRISMA flow diagram.

Based on a gap identified concerning how PCC and QoL are interrelated where a qualitative synthesis of information would support further understanding, a systematic literature search (Figure 1; Supplementary Material) was conducted in January 2020 and again in March 2021 using the following databases: CINAHL (EBSCO), PubMed (EBSCO), PsycINFO (Proquest), and SCOPUS. The searches comprised three search blocks including variants of (a) Long-term Residential Care, (b) Quality of Life/Well-being, and (c) Person-Centered Care. Boolean operators AND and OR were used to combine search terms. Phrase searches, proximity operators, and truncation were also used. All terms were searched by title and abstract. Controlled vocabulary terms were used when provided by the database. Search terms were decided using suggestions from current literature, Cochrane published reviews, pilot searches, and discussions among the research team. Studies were eligible for inclusion if they included insight into QoL or well-being in LTRC or any aspects professing to contribute to QoL, such as personhood in LTRC. LTRC had to offer PCC, or a variant of PCC to be included. The transition into LTRC could be finalized or ongoing to be eligible for inclusion, with no restriction on length of stay where the transition had been finalized. Primary studies using qualitative or mixed-methods designs were included in the final selection. Where mixed-methods designs were used, only qualitative results were included. Studies had a target population of LTRC residents aged 65 or older, which could also include those living with cognitive decline or dementia. The information reporting on QoL, experiences of QoL, or any aspect of health-related QoL could have been self-reported, reported by proxy, or observed by a third party, for example, relatives or staff members. All inclusion criteria were agreed among the research team ahead of the screening process. All screening (title/abstract and full text) was undertaken independently by two reviewers (100% dual-screened, the first and last authors), and any discrepancies were reviewed by two independent reviewers (see Table 1 for included study characteristics).

**Table 1.** Study Characteristics

Study	Country	Methodology	Method	Sample	Aim
**Edvardsson et al., 2010**	Australia	Qualitative	Interviews (individual/focus groups/telephone)	Persons living with dementia (*n* = 11), staff (*n* = 37), family (*n* = 19)	To describe the content of PCC as described by persons living with dementia, family members, and staff in LTRC
**Barnes et al., 2013**	UK	Qualitative	Observations	Residents (*n* = 68) in four LTRC homes	Describing individual residents’ mealtime experience to understand best practice
**Adra et al., 2015**	Lebanon	Qualitative	Semistructured interviews	Residents (*n* = 20), staff (*n* = 11), family (*n* = 8)	To describe and explore QoL
**Nakrem, 2015**	Norway	Qualitative	Ethnographic observation and interviews	Observations: LTRC (*n* = 4), resident interviews (*n* = 16; selected from the four LTRC homes)	To describe LTRC culture from staff perspective including how residents describe QoC
**Williams et al., 2015**	Canada	Mixed-methods	Interviews (focus group/individual)	Staff (*n* = 19)	To evaluate staff experiences of an implemented PCC program and resident outcomes
**Donnelly et al., 2016**	Canada	Qualitative	Interviews and observations	Residents (*n* = 21)	Resident perceptions of care in LTRC offering PCC
**Hartmann et al., 2018**	USA	Mixed-methods	Observations and interviews	Staff interviews (*n* = 66), resident and staff observations (*n* = 1,490)	To describe how elements of PCC can improve resident engagement
**Baxter et al., 2019**	Australia	Qualitative	Narrative interviews	Residents (*n* = 21)	To illuminate meanings of thriving as narrated by persons living in nursing homes
**Helgesen et al., 2020**	Norway	Qualitative	Focus group interviews	Staff (*n* = 21)	To elicit health care staff experiences of implementing one-to-one contact between residents and care staff in nursing homes
**Hennelly et al., 2021**	Ireland	Qualitative	Semistructured interviews	Persons living with dementia (*n* = 8), family (*n* = 8), staff (*n* = 15)	To generate an understanding of current approaches to “personhood”

*Note:* PPC = person-centered care; LTRC = long-term residential care; QoL = quality of life; QoC = quality of care.

### Data Extraction and Analysis

#### Phase 3

Each included study was read in full multiple times to obtain a full picture of the phenomenon. During this time, definitions of PCC were determined from each study and tabulated (Table 2), and a list of potential themes and potential relationships between studies were established. This list was reduced into relevant categories. Minor discrepancies in paper or theme inclusion were resolved during discussion within the research team.

**Table 2.** Definitions of Person-Centered Care and Variants

Author	Term used	Definition based on author understanding/research
Edvardsson et al., 2010	Person-centered care	Promoting a continuation of self and normality
Barnes et al., 2013	Resident-centered care	Fitting activities to the individual needs of the resident, providing support while fostering independence
Adra et al., 2015	Relationship-centered care	Negotiations that consider the needs of everyone involved, as well as the context of the wider community. Resident, staff, and family contributions are viewed with equal importance
Nakrem, 2015	Person-centered/relationship-centered care	Adopting individual resident perspective and recognizing resident/family values. Developing a shared understanding of the needs and values of residents, staff, and family members
Williams et al., 2015	Person-centered care	Residents should be understood by individual needs, preferences, abilities, and life experiences
Donnelly & MacEntee, 2016	Person-centered care	Placing the resident and their individual needs and preferences at the center of care
Hartmann et al., 2018	Collaborative care	Providing an integrated care system
Baxter et al., 2019	Person-centered care	Involving residents in decision making and encouraging resident independence
Helgesen et al., 2020	Person-centered care	An ideal—a means of preserving a vulnerable person’s dignity and well-being
Hennelly et al., 2021	Person-centered care	Care elements of personhood: interests; preferences; life course experiences; social interaction; family; and place should all be included in person-centered care models

During analysis, outcomes were discussed within the review team. Line of argument synthesis was developed during reflective discussion of each construct using perspectives of the whole review team; a constructivist approach as shown in the phases outlined below was used to achieve this (Atkins et al., 2008; Britten et al., 2002; Noblit & Hare, 1988). The review structure follows the guideline Enhancing Transparency in Reporting the Synthesis of Qualitative Research (Tong et al., 2012).

### Meta-Synthesis and Analysis

#### Phase 4: Identifying relationships across the studies

Using the information presented in the results, discussion, and conclusion sections of the included papers, we were able to establish author interpretations relating to PCC and QoL. During this time, we constructed themes, which were then used to determine relationships across the studies, which formed our second-order constructs (Table 3; table 4).

**Table 3.** Second-Order Construct Inclusion by Study

Study	Themes				
	Maintaining independence, dignity, and autonomy	Knowing the whole person	Creating a “homelike” environment	Establishing a caring culture	Integrating families and nurturing internal and external relationships
Edvardsson et al., 2010	Residents should be acknowledged and respected as competent/valuable people	Knowing each individual resident history important to staff	Important to enrich environment with personal items and suitable aesthetics	Residents should be prioritized over tasks	Family involvement is important to all actors, but in different ways
Barnes et al., 2013	Different settings can promote resident independence. Choice improves residents’ experience	Understanding needs/limitations important to personalized care	N/A	Staff had good knowledge of resident care requirements	Level of interaction is dependent on the setting. Residents requiring more support have the most interaction
Adra et al., 2015	Listening to residents and providing “meaningful activities” improve dignity	N/A	Personalized space integral to PCC and resident QoL	N/A	Family/friends still play valuable roles. Continuity from new relationships between residents
Nakrem, 2015	N/A	Maintaining personal routines important	Personalized space provides continuity	PCC = “continuous learning process”	N/A
Williams et al., 2015	Need to balance promoting independence/creating risk	QoL improved by knowing the resident history	N/A	Different facilities focused on different elements of PCC	N/A
Donnelly & MacEntee, 2016	Asking for help should be encouraged. Individual limitations/needs must be understood. Choice provides autonomy	Base care on individual needs/ preferences	N/A	Residents viewed task- based care negatively	N/A
Hartmann et al., 2018	N/A	Knowing individual resident needs is important to staff. Affected by staff turnover	Personalized space promotes meaningful conversations	N/A	N/A
Baxter et al., 2019	Choice and continuity support independence and help residents “thrive”	Care based on needs/choices improves resident well-being/ QoL	Good atmosphere as important as homelike living space	Care/support in LTRC = “knowing someone’s there”	Resident–staff relationships provide further social support
Helgesen et al., 2020	N/A	Knowing residents = positive for staff/residents. Some residents prefer short interactions/decline 1:1 contact	Environment can facilitate/prevent 1:1 contact	1:1 time outside of necessary care should be standard practice. More 1:1 time = calmer unit	1:1 time with residents provides additional social support
Hennelly et al., 2021	Resident choice is important for personhood. Supporting independence helps persons living with dementia live well	N/A	N/A	Power imbalances/ permissions block autonomy	Family = either positive or negative depending on nature of relationship

*Note:* PPC = person-centered care; LTRC = long-term residential care; QoL = quality of life.

**Table 4.** Definitions of First-, Second-, and Third-Order Constructs

Term	Definition
First-order construct	Participant views and beliefs as outlined in primary studies
Second-order construct	Author interpretation of participant views and beliefs within primary studies
Third-order construct	Reinterpretation of explanations offered based on first- and second-order constructs, providing overarching themes/metaphors

*Note:* From Purc-Stephenson & Thrasher (2010).

#### Phase 5: Translating papers into each other

Following the tabulation of second-order constructs, reciprocal translation was used to identify “commonalities and contradictions” in the included studies to synthesize the information and build on it from the perspectives of the research team (Dixon-Woods & Fitzpatrick, 2001; France et al., 2019). Themes concluded from this meta-synthesis were constructed from synonymous concepts found across the included studies to help determine potential interrelations existing between PCC and QoL following a permanent transition into LTRC, which were discussed in the research team in order to be reinterpreted (Grant & Booth, 2009; Ring et al., 2011).

#### Phase 6: Synthesizing translations

Themes were reinterpreted in a second level of synthesis to provide explanation, forming the third-order constructs presented in the results section. The second-order constructs were discussed within the research team and, using our different backgrounds, were elaborated on to form a new conceptual understanding and arguments for the interrelating factors between PCC and QoL in LTRC and demonstrated new interpretations of the second-order constructs.

#### Phase 7: Presenting the synthesis

The synthesized translations were presented as (a) personalizing care within routines, (b) optimizing resident environments, and (c) giving residents a voice.

## Results

About 969 potentially relevant studies were identified following database and hand searches. In that 61 studies were screened by full text, of which 10 met the inclusion criteria for this review.

### Second-Order Constructs

Qualitative data from seven countries were found in the 10 primary studies included, which indicated the following second-order constructs: (a) maintaining independence, dignity, and autonomy; (b) knowing the whole person; (c) creating a “homelike” environment; (d) establishing a caring culture; and (e) integrating families and nurturing internal and external relationships. Table 3 presents the occurrence of the second-order constructs in each study, the detail of which is discussed fully in the following sections.

### Maintaining Independence, Dignity, and Autonomy

Ordinarily, a move into LTRC follows a need for additional care or support. However, regardless of the level of dependence of a person, they remain a person of value and should be respected and treated with dignity. In order to provide continuity during a move, residents should be provided with choice, and the need for care should be balanced with supporting independence. This is integral not only to achieving PCC in LTRC, but also in maintaining resident QoL (Barnes et al., 2013; Baxter et al., 2019; Edvardsson et al., 2010; Hennelly & O’Shea, 2021).

Provision of care and support in LTRC creates a feeling of safety and ensures physical/functional needs are addressed; however, support should go beyond physical care to provide residents with a level of choice to maintain a sense of self and increase personhood and QoL (Barnes et al., 2013; Baxter et al., 2019; Williams et al., 2015). Individualizing care and considering both physical and behavioral resident needs could better maintain independence, which promotes resident QoL (Donnelly & MacEntee, 2016; Williams et al., 2015). It is important for staff to understand individual resident limitations and needs to simultaneously provide adequate care and create an environment where residents feel comfortable asking for additional help, while also encouraging residents to take charge of their own care/activities where possible to support independence (Barnes et al., 2013; Donnelly & MacEntee, 2016). Overestimating a resident’s ability could put the resident at risk, for example, of falls; therefore, the level of support required by each resident must be acknowledged while encouraging independence (Edvardsson et al., 2010; Williams et al., 2015). Staff limitations and time constraints should also be recognized, as organizational needs can dictate staff behaviors and put pressure on staff. This can reduce the possibility of staff–resident interactions, making it difficult for staff to promote resident independence (Adra et al., 2015).

Sensitivity to privacy and dignity is at times overlooked in residents requiring more assistance with daily living activities. Resident interviews emphasized this, discussing times when they were “wheeled down the hall half dressed” or when they felt their voice was overlooked, affecting dignity (Donnelly & MacEntee, 2016). Residents expressed a desire to be listened to rather than being “talked over,” particularly during activities or when relatives were present to improve personhood and prevent them feeling they were being treated like children, which is how some residents recounted the experience (Adra et al., 2015; Hennelly & O’Shea, 2021).

Organizational needs, policies, and staff demands often cause rigid routines within LTRC, which residents felt meant they were all subject to the same routine, reducing the possibility of resident choice and QoL (Barnes et al., 2013; Baxter et al., 2019; Donnelly & MacEntee, 2016; Nakrem, 2015). Regardless of staff limitations or the level of dependency of individual residents, it is important to provide flexibility and choice in care wherever possible in order to successfully achieve PCC, maintain resident QoL, and allow residents to thrive in LTRC rather than simply surviving (Barnes et al., 2013; Baxter et al., 2019; Hennelly & O’Shea, 2021).

### Knowing the Whole Person

Following a move to LTRC, there is a risk that a resident will lose their sense of self; therefore, a comprehensive history of the resident should be taken, which includes medical and biographical information as well as likes and dislikes of the resident. This assists with a smoother transition into care and promotes resident QoL (Adra et al., 2015; Edvardsson et al., 2010; Williams et al., 2015).

Getting to know a new resident as a “whole” person should incorporate information from the resident as well as family members or close friends who know the person best, which follows the concept of PCC (Adra et al., 2015). Taking time to learn resident histories, needs, interests, and preferences is important to be able to engage residents in meaningful conversations and activities and personalize routines, all of which contribute to resident QoL (Barnes et al., 2013; Donnelly & MacEntee, 2016; Edvardsson et al., 2010; Hartmann et al., 2018; Williams et al., 2015). The difficulty is that presence and availability of staff is key in facilitating informal conversations with residents outside of a care routine (Edvardsson et al., 2010). In an intervention to spend more one-to-one time with residents, staff immediately found getting to know residents better to be a positive experience (Helgesen et al., 2020).

Increased staff–resident engagement enables staff to get to know residents better, allowing them to have better-quality interactions and adapt activities to suit different residents, making them more meaningful (Edvardsson et al., 2010; Hartmann et al., 2018; Nakrem, 2015). This was found to not only help maintain resident sense of self, but staff also found this made the working day easier as such quality interactions had a calming influence on residents, and the setting as a whole, including for persons living with dementia (Hartmann et al., 2018). Times when staff were viewed by residents as preoccupied or burdened were described by residents as making them feel less engaged with the care team (Adra et al., 2015; Donnelly & MacEntee, 2016; Edvardsson et al., 2010; Hartmann et al., 2018).

Getting to know a resident well and including them in the care planning process enables PCC. Regular one-to-one contact with residents beyond care delivery was integral to achieving PCC (Helgesen et al., 2020). Highlighting the need for such interactions helped staff become more aware of low activity and encouraged them to provide additional stimulation for residents (Hartmann et al., 2018). In fact, they found that this culture change spread beyond those involved in the intervention and uptake was seen in the majority of staff, which was seen as a positive step. However, Donnelly and MacEntee (2016) found that according to residents, imposing activities on residents under the assumption they should be stimulated led to a loss of autonomy, which reduced QoL.

### Creating a “Homelike” Environment

Residents, staff, and family members mutually agree that the ability to personalize resident living space following a move into LTRC is integral to PCC and resident QoL. Allowing the resident to create an environment reminiscent of the home they moved from, provides familiarity and continuity during a transition to LTRC, which is particularly important to maintain QoL for persons living with dementia (Adra et al., 2015; Baxter et al., 2019; Edvardsson et al., 2010; Nakrem, 2015).

Residents felt it important to have “normal” things around them to remind them of their life before moving into LTRC and to provide them with a personalized space. Personalizing a space with “homelike” items, such as photos, pictures, plants, and furniture, allows the resident to show their personality as well as providing continuation of self and a sense of normality for the resident, which is key to achieving PCC (Adra et al., 2015; Edvardsson et al., 2010). In addition to enriching the environment, personalizing resident space facilitates staff-resident contact by providing conversation topics, which provides content for more meaningful conversations leading to improved resident QoL (Edvardsson et al., 2010; Hartmann et al., 2018; Helgesen et al., 2020).

In addition to individual resident rooms, it is important to consider the aesthetics in open communal spaces providing access to mutual activities, such as gardening. Creating a welcoming atmosphere can encourage social relationships between residents and encourage residents to continue a past hobby (Adra et al., 2015; Edvardsson et al., 2010). Continuation of self is enhanced when in flexible surroundings, which goes beyond resident bedrooms. A sense of “home” is enhanced by providing views of and easy access to outside space (Edvardsson et al., 2010). The whole environment and overall aesthetics in LTRC should be welcoming and comfortable for residents, providing an open but safe atmosphere (Baxter et al., 2019). Clear and calming decoration, in particular, helps persons living with dementia to settle into life in LTRC (Edvardsson et al., 2010).

### Establishing a Caring Culture

Culture change in LTRC is a “continuous learning process,” which is dependent on adequate communication and training among staff. Even with training, what PCC should entail can look differently across teams, with each individual focusing on different core elements of PCC (Nakrem, 2015; Williams et al., 2015).

Successful PCC should centralize resident needs and prioritize residents over tasks. Baxter et al. (2019) explored the possibility for a resident to “thrive” in LTRC and found that each aspect contributing to this proved a “one-size-fits-all” approach does not work. However, data in other included papers suggest that structural and cultural differences between LTRC homes mean this is not always possible (Edvardsson et al., 2010; Nakrem, 2015). In interviews, residents occasionally observed staff as being too task-focused, which residents expressed as causing dissatisfaction with their care (Barnes et al., 2013; Donnelly & MacEntee, 2016). Observed instances of staff able to interact more freely with residents during tasks seemingly improved resident experience and QoL, and staff expressed a reduction in guilt (Barnes et al., 2013; Helgesen et al., 2020). In interview data presented, residents praised the level of support offered by staff during these prolonged interactions; although the same data also demonstrated that simply knowing someone is there and feeling able to ask for help, even someone external to the LTRC home such as a family member, GP, or volunteers increases feeling of support (Baxter et al., 2019; Donnelly & MacEntee, 2016). Occasionally, organizational needs and rigid structures in place could not be changed and acted as a barrier to achieving PCC, causing residents to express dissatisfaction (Helgesen et al., 2020; Nakrem, 2015). Although Nakrem (2015) actually found that a certain level of routine or some rhythm to the day provided residents with a feeling of safety and being well cared for.

Good communication across all actors is key to successfully implementing PCC. It has been found that differing views among the care team or between family and LTRC staff, particularly involving persons living with dementia, can create a barrier to supporting personhood and providing PCC (Donnelly & MacEntee, 2016; Hennelly & O’Shea, 2021). Differing opinions between staff and relatives surrounding care can affect resident personhood and create an additional barrier for PCC (Hennelly & O’Shea, 2021).

### Integrating Families and Nurturing Internal and External Relationships

A common ground among different variants of PCC in LTRC in the included studies is the involvement of multiple actors, including family members, various staff, volunteers from the local community, and the resident themselves.

Often, care provided before a permanent move into LTRC is provided by family members or close friends, which Adra et al. (2015) found did not immediately end following a move into LTRC. In addition, the involvement of family in care positively affected outcomes for both residents and family members. Family members interviewed felt their inclusion in the life and care of their resident was an integral part of achieving PCC, which staff agreed with as knowledge provided by family members assisted with care planning and supported resident QoL (Adra et al., 2015; Edvardsson et al., 2010). For residents and family members, this integration also helped with adjusting to change. Family members could remain a significant part of the resident’s life, while residents value continuation of “normal life” during the transition into LTRC; both of which are particularly important for persons living with dementia (Adra et al., 2015; Edvardsson et al., 2010).

Family involvement following a move into LTRC could be a positive experience, providing continuity and support, or a negative experience, creating anxiety for persons living with dementia and staff depending on how the relationship was prior to the move and the understanding of dementia (Hennelly & O’Shea, 2021). Family members emphasized the importance of communication, which reduces their own anxiety about a resident, where staff described the opportunities for teamwork between staff and family members. Poor communication between staff members and relatives can create interpersonal barriers, which prevents personhood in persons living with dementia (Hennelly & O’Shea, 2021). Overall, family input is regarded in the studies as beneficial; however, residents highlighted that it should not cause them to feel like they are being “overlooked” (Donnelly & MacEntee, 2016). Encouraging and incorporating family in care provides additional support for residents, while reducing the burden on family members, which helps achieve PCC and maintain resident QoL (Adra et al., 2015; Barnes et al., 2013; Hennelly & O’Shea, 2021). Interview data demonstrated that creating opportunities for interactions with family members is integral to “thriving” in LTRC. In addition, interactions with friends and the wider community were also found to provide “a sense of social support and connection” (Edvardsson et al., 2010).

Resident interactions with relatives, staff, and other residents are important to create a feeling of connectedness and support for residents, but should be led by the resident. However, interactions can be encouraged by all parties within the LTRC environment and are important to achieve PCC and maintain resident QoL (Barnes et al., 2013; Baxter et al., 2019; Edvardsson et al., 2010). New relationships between residents within LTRC were seen by residents as providing continuity between past and present circumstances; however, encouraging resident interactions was not always welcome, with residents expressing and observed as sometimes preferring solitude (Adra et al., 2015; Baxter et al., 2019). In addition to resident interactions, informal interactions with staff were also described in interview data as improving resident moods (Baxter et al., 2019). Staff interviewed echoed the value of this one-to-one interaction in a different study (Helgesen et al., 2020). In general, residents felt opportunities for informal interactions were important; although it is important that the level of interaction be decided by the resident (Baxter et al., 2019).

### Synthesizing Translations

After synthesis of the second-order constructs, the following third-order constructs emerged and were developed: (a) *personalizing care within routines,* (b) *optimizing resident environments, and* (c) *giving residents a voice*.

### Personalizing Care Within Routines

It is clear from the included papers that an understanding of the importance of personalized care in achieving PCC exists to provide continuity for residents and more control over their daily lives in LTRC. The difficulty is, while the included papers agree that personalized care is optimal, they also demonstrate clear barriers to implementing it. Unavoidable routines and restrictions imposed on LTRC staff are eluded to, preventing personalized care from being carried out in the way it is fully intended. These routines are discussed throughout included papers as a significant barrier to PCC, as they prevent shared decision making, restrict resident autonomy, and create a focus on task completion rather than residents. If this is the case, LTRC risks feeling less personalized, which threatens resident QoL. It must be recognized that perceptions of routines within the included papers largely come from LTRC staff, so it is difficult to know whether the routines are as rigid as described or whether this is perceived organizational control.

In addition to preventing PCC, imposed routines are said to reduce staff–resident engagement beyond daily care needs, which makes it more difficult to promote resident autonomy and independence. Some residents’ found routines provided structure resulting in a sense of safety, although mostly the inability to change routines within LTRC was negatively discussed or residents were resigned to it, but not happy about it. It should be stressed that routines were imposed from an organizational standpoint and were simply implemented by the staff, who in most interview extracts also saw this as a negative element of the care provided.

Residents moving into LTRC must adapt from independent living to a regulated community environment, which will undoubtedly alter their usual routine. There will always be restrictions to providing a fully personalized routine, for example, it is difficult to serve meals at staggered times when catering to a large number. However, by providing lunch options including different portion sizes for residents to choose from begins to recentralize residents in the activity. Small steps such as discussing with a resident whether they prefer to shower in the morning or evening, what time they like to get up and go to bed, and how they prefer to structure their day around activities that are difficult to change immediately increases resident autonomy and eases the transition into LTRC.

### Optimizing Resident Environments

Personalizing resident environments is discussed as a key element in PCC, with staff and resident interviews in the included studies showing that moving with personal items, such as furniture and photographs, as well as tailoring the general aesthetics to each resident eases the transition into LTRC and improves resident QoL. This is considered to be particularly important for people living with either cognitive decline or dementia, for whom this familiarity can provide continuity, comfort, and an identity following a transition into LTRC. However, potential limitations such as physical needs of a resident or organizational policy can make implementation of all desired personalized changes more difficult. For example, if a resident moves into LTRC needing specialist equipment, it may not be feasible to bring furniture they previously used when living in the community. Although, providing equipment to meet the individual needs of a resident is also integral to PCC. Within resident rooms, it should be possible to provide a blank canvas other than required specialist equipment so that a resident can personalize them as much as they wish.

It is clear from included papers that optimizing resident environments goes beyond individual rooms. The whole environment should be considered a resident’s home as would be the case in any communal living environment. Personalizing communal spaces to suit the preferences of each resident is challenging; however, discussing design with residents can give an idea of preferred aesthetics and includes residents in the decision-making process. Providing spaces that residents can contribute to, such as gardens or wall art, could allow residents to feel more at home and part of the community as well as encouraging independence and personhood. Having the right aesthetics can contribute to residents feeling welcome and at ease following a transition into LTRC.

Creating space for residents to continue hobbies outside of prearranged activity groups, such as gardening, could improve personhood and act as a conversational focus between residents or residents and staff, therefore increasing interaction. This provides a space to explore shared hobbies in a less structured way. In addition, communal spaces, such as dining areas, could increase the opportunity for resident interaction depending on the layout.

### Giving Residents a Voice

Knowing the resident, including a biographical history, their likes, and dislikes (past and present), as well as their medical and daily care needs, enables PCC and creates continuity during a transition into LTRC. It is important that resident histories, likes, and dislikes come primarily from residents. While family members can provide valuable insights into who the resident was and who they are now, it is important that a resident is also able to share this information. In fact, some studies in this review show that the focus of information given often differs depending on whether it comes from family or the resident; for example, family will focus more on physical aspects, such as diet, where residents focus on social aspects such as likes and dislikes. There is a risk of overlooking the resident in favor of relative viewpoints and wishes, which should be avoided to achieve PCC.

Knowing the person should incorporate who the resident is today as well as their history. This is particularly important for persons living with dementia, who in some cases have forgotten what their past preferences were, but may be able to communicate what they enjoy today. This concept is often overlooked and an importance tends to be placed on knowing who the person “was” rather than seeing them as the person they are today. In this case, it is important that perspectives of both residents and family are brought in to listen to the resident preferences today, as well as those expressed in the past to create a whole picture.

In order to really “know the person,” it is also important to consider how they feel following a transition into LTRC. It is common for older people to experience a sense of loss during such a transition, whether this is a result of leaving people, or the environment they have been used to living in. It is important for staff to understand and help residents to navigate this by talking and listening to residents. Information obtained to get to know the resident should be translated into shared decision making relating to care and routines wherever possible in order to facilitate PCC and improve QoL. A focus on resident experiences makes it possible to understand necessary changes in care or routines, implement PCC, and improve QoL.

## Discussion and Implications

The results of this review indicate that implementing a person-centered model of care enhances resident QoL, although exactly what PCC means differs across the included studies. The synthesized data suggests that enabling the personalization of care and resident environments and ensuring resident voices are heard are strong interrelating factors between PCC and QoL. Lack of adaptability within LTRC creates a barrier to implementing PCC, compromising resident autonomy and independence, which ultimately reduces resident QoL. Simple changes within LTRC applied to routines and the environment considering the voice of the residents facilitates PCC and improves QoL.

A major barrier to achieving PCC in LTRC observed in this review is restrictions imposed by organizationally influenced routines. This is not a new concept, with previous studies hearing from staff that even when PCC is there, time forces a task-orientated approach to care (Oppert et al., 2018). It is therefore important to focus on flexibility in routines to support resident independence and autonomy, which is beneficial to people with cognitive decline (Kane et al., 2004; Oppert et al., 2018). The difficulty is that PCC is being implemented alongside a number of barriers, such as lack of time, staff or money, or even too many residents (Kong et al., 2022). It is this attempt to implement PCC without understanding local contexts, which was outlined by Berntsen et al. (2019) as a gap in clearly understanding how elements of PCC can be put into practice. It is important therefore to look at how organizational and personalized routines can complement each other to benefit resident QoL, for example, by altering serving styles at mealtimes as indicated by Barnes et al. (2013) rather than adding further pressure to staff by suggesting fully moving to personalized care for all. Manageable personalization of routines, such as individual wake-up times and providing choice of times for daily care, can introduce elements of PCC, while respecting necessary organizational routines such as meal times, which are more challenging to alter. It is important to consider such organizational factors while also working to improve resident experiences (Rahman & Schnelle, 2008). By doing this, the physical health, environmental, and psychological health elements of the WHO QoL domains could be achieved.

Staff–resident interaction, allowing staff to get to know residents better, can be facilitated or obstructed by the LTRC home environment according to Helgesen et al. (2020). This, according to WHO (2019), is an important QoL domain “social relationships.” Providing personalized resident environments has been suggested as a key element in achieving PCC in previous research, as it provides continuity during a transition to LTRC (Bradshaw et al., 2012). The findings of this review support this, showing that familiar and flexible surroundings support a continuation of self for the resident, which improves personhood and QoL (Edvardsson et al., 2017). Furthermore, the results of this review indicate that environment goes beyond resident bedrooms and should include communal spaces, which are key to encouraging resident–resident and resident–staff social interactions by providing talking points and access to shared interests (Adra et al., 2015; Edvardsson et al., 2017).

Getting to know residents well as they move into LTRC helps staff to understand personal preferences that can be met, which helps to achieve PCC by individualizing routines as much as possible within organizational constraints (Baxter et al., 2019; Hennelly & O’Shea, 2021; Nakrem, 2015). Previous quantitative research has found that within LTRC that has fully implemented PCC, resident choice and staff knowledge of resident preferences are associated with resident satisfaction and higher resident QoL (Poey et al., 2017). The qualitative data within this review support this and additionally suggest that when staff are able to get to know residents well, it is possible to make activities more meaningful. This not only increases residents’ sense of self, but also makes things easier for staff, who also commented on the benefits of knowing their residents (Edvardsson et al., 2010; Hartmann et al., 2018; Helgesen et al., 2020).

Integrating family into LTRC was suggested in this review as a way of getting to know the resident further and enabling the implementation of PCC. The results of this review particularly highlighted the importance of integrating family members into both the care planning process and the LTRC environment to facilitate PCC (Adra et al., 2015; Barnes et al., 2013; Edvardsson et al., 2010; Hennelly & O’Shea, 2021). Past quantitative research exploring perceived resident QoL from the perspective of family members found that resident QoL was perceived as higher when communication between family members and staff was strong (Roberts & Ishler, 2017). Furthermore, Dewing and McCormack (2017) stated that a strong collaboration between multiple actors, which includes family members, is key to a successful PCC culture in LTRC. However, results in this review from a resident perspective suggest that staff–family communication can at times overshadow the voice of the resident, causing them to feel overlooked and risking their autonomy (Donnelly & MacEntee, 2016). Supporting resident autonomy is key in providing PCC and maintaining resident QoL; therefore, residents should have a voice in how family are integrated into LTRC. Although, it is important to explore what integrating family members into LTRC could mean for the resident and how best to achieve this from the perspective of the resident as well as the family member.

The findings of this review indicate that there are still barriers to implementing PCC, which has previously been said to risk the belief that PCC has been fully implemented, when it in fact has not (Dewing & McCormack, 2017). At an organizational level, perceived barriers, such as time constraints, prevent PCC from being implemented in full, which Dewing and McCormack (2017) explain can create feelings of guilt and failure among staff. At an individual level, the key interrelating factor between PCC and QoL is the resident feeling like they have a voice and choice, which includes adding personalized elements to routines and allowing them to feel at home within the environment. Therefore, in reality, there are aspects of PCC that can be implemented around organizational constraints, which would maintain resident QoL without being so complex that extra pressure is put on staff. However, organizational constraints within each LTRC home need to be considered that change depending on context. Culture, physical building design, and governing bodies alter which PCC elements can be implemented within each LTRC home. By creating a realistic PCC culture in LTRC, which puts emphasis on strengthening communication with residents and family members and recognizes the elements of routine and environment that can be personalized, the key interrelating factors contributing to PCC and QoL can be achieved.

### Strengths and Limitations

The applied meta-ethnographical approach enabled previous findings based on inductive qualitative research relating to PCC and QoL from heterogeneous contexts not only to be synthesized, but to be reinterpreted with insight from each member of the interdisciplinary team. This procedure gave additional value to individual qualitative studies by translating them into each other, therefore allowing us to go beyond a comparison to fully explore relevant interrelating factors in varying cultural contexts of LTRC.

A limitation in this review is that without access to the original data analyzed within each study, any reinterpretation of data was limited to what was presented in each paper, allowing only selected data to be discussed and reinterpreted. The final number of papers eligible for inclusion in this review was small in number, so we were unable to filter papers based on the richness of data, which has been suggested by France et al. (2019) as a method to improve meta-ethnographic reporting. Therefore, the data included in this review are not equally rich across all studies. Furthermore, the background of each study has not been included in detail when creating the second- or third-order constructs, which means that the detailed description of context normally important in qualitative studies had to be somewhat overlooked. However, the use of reciprocal translation within this review allowed us to focus on commonalities and differences across studies to provide novel and inductively grounded insights to develop a consistent interpretive synthesis (Dixon-Woods & Fitzpatrick, 2001; Noblit & Hare, 1988).

## Conclusion

There are many interrelating aspects of PCC and QoL following a permanent transition into LTRC, but how PCC can be and is performed still presents challenges. There is a clear need for good communication across multiple actors to successfully implement PCC, but it is important to prioritize resident voices to get to know residents well so that their needs and preferences can be factored into care planning and organizational routines. In doing this, QoL will be supported and the transition into LTRC will be a more positive experience. Future research should seek to understand how these key interrelating factors can be implemented while considering context to understand exactly what is possible.

## Supplementary Material

gnac027_suppl_Supplementary_Material
